# Systematic review and meta-analysis of clinical trials: In–hospital use of sensors for prevention of falls

**DOI:** 10.1097/MD.0000000000027467

**Published:** 2021-10-15

**Authors:** Olga L. Cortés, Hillary Piñeros, Pedro Antonio Aya, Jefferson Sarmiento, Indira Arévalo

**Affiliations:** a Department of Research and Department of Nursing. Fundación Cardioinfantil - Instituto de Cardiología, Bogotá, Colombia; b Biomedical Engineering student, Faculty of Biomedical Engineering. Universidad del Rosario - Escuela Colombiana de Ingeniería Julio Garavito, Bogotá, Colombia; c Biomedical Engineer, MSC. Faculty of Biomedical Engineering. Universidad del Rosario - Escuela Colombiana de Ingeniería Julio Garavito, Bogotá, Colombia; d Electronic Engineer, Faculty of Biomedical Engineering. Universidad del Rosario - Escuela Colombiana de Ingeniería Julio Garavito, Bogotá, Colombia; e Nurse, Director of Nursing Department. Clínica Universidad de la Sabana, Chía, Cundinamarca, Colombia.

**Keywords:** elderly, fall prevention, inpatient, monitoring, sensors, technology

## Abstract

**Background:**

Intra-hospital falls have become an important public health problem globally. The use of movement sensors with alarms has been studied as elements with predictive capacity for falls at hospital level. However, in spite of their use in some hospitals throughout the world, evidence is lacking about their effectiveness in reducing intra-hospital falls. Therefore, this study aims to develop a systematic review and meta-analysis of existing scientific literature exploring the impact of using sensors for fall prevention in hospitalized adults and the elderly population.

**Methods:**

We explored literature based on clinical trials in Spanish, English, and Portuguese, assessing the impact of devices used for hospital fall prevention in adult and elderly populations. The search included databases such as IEEE Xplore, the Cochrane Library, Scopus, PubMed, MEDLINE, and Science Direct databases. The critical appraisal was performed independently by two researchers. Methodological quality was assessed based on the ratings of individual biases. We performed the sum of the results, generating an estimation of the grouped effect (Relative Risk, 95% CI) for the outcome first fall for each patient. We assessed heterogeneity and publication bias. The study followed PRISMA guidelines.

**Results:**

Results were assessed in three randomized controlled clinical trials, including 29,691 patients. A total of 351 (3%) patients fell among 11,769 patients assigned to the intervention group, compared with 426 (2.4%) patients who fell among 17,922 patients assigned to the control group (general estimation RR 1.20, 95% CI 1.04, 1.37, *P* = .02, I^2^ = 0%; Moderate GRADE).

**Conclusion:**

Our results show an increase of 19% in falls among elderly patients who are users of sensors located in their bed, bed-chair, or chair among their hospitalizations. Other types of sensors such as wearable sensors can be explored as coadjutants for fall prevention care in hospitals.

## 1 Introduction

The World Health Organization (WHO)^[1]^ defines the term fall as the consequence of any event that hurls an individual toward the ground against his/her will. Accidents constitute the sixth cause of death in people over 65 years of age, and falls are the principal cause of accidental death, especially in older adult individuals, whether preventable or not preventable. This is the most notified sentinel event in the Joint Commission Database (USA).^[2]^ Intra-hospital falls have become an important public health concern globally.^[3]^ Falls constitute 38% of adverse hospital events. Approximately 30% of intra-hospital falls result in lesions and approximately between 4% and 6% result in serious lesions, such as fractures, subdural hematoma, bleeding, and death.^[3,4]^

For health provider institutions, a fall-type adverse event means the loss of the levels that accredit it as an institution of quality by questioning the safety of their patients.^[5]^ The weight of the problem is expressed in the increased cost to health system.^[1,2]^ According to the Center for Disease Control (CDC),^[2]^ the cost of falls was around $19-billion US dollars in 2007 globally, and it is expected that by 2020 the annual cost will increase by $54.9-billion US Dollars if no evidenced-based strategies are implemented to reduce the rate of these and secondary lesions.^[6]^

Falls of high risk patients in hospital are associated, among others, with environment factors such the existence of inadequate flooring, lighting problems in the rooms, presence of obstacles in the rooms, complete use of guardrails that limits movement, along with lacks of communication systems between patients and their caregivers. The majority of falls occur in patients’ rooms and constitute more than half of all the falls. Other habitual places are the hallway outside the room and the bathroom in room.^[6]^

Given these factors, scant attention has been paid to research on the use of intra-hospital technological and bioengineering tools, as adjuvants in the preventive medical/nursing care plan for patients with high risk of falling.^[7–10]^

The installation of call bells and alarms in the rooms of hospitalized patients alerts caregivers about patients’ needs of movement that demand help.^[9–11]^ Nevertheless, the use of this light or bell alarm system operated by the patient is questioned in fall prevention, especially when the patient does not communicate to move due to health difficulties; therefore, changes in position and initiation of the walk are not detected on time by nursing prior to the fall.

The use of movement sensors with alarms has been studied as elements with a predictive capacity for falls at hospital level.^[7–10]^ These are offered to caregivers as tools installed on beds, chairs (pressure sensors), or less frequently adhered to patients (portable accelerometers-gyroscopes), with the capacity to predict falls in hospitalized patients. However, despite their use in some hospitals throughout the world, evidence is lacking about their effectiveness in reducing intra-hospital falls.^[4,5,7]^

Bearing in mind this uncertainty, we explored the following question: Is there any impact on falls? among adult patients receiving preventive care with sensor-based devices placed on elements of hospital use or portable during hospitalization?

## 2 Methods

### 2.1 Objective

The principal aim was to perform a systematic review of scientific literature available by exploring the impact of using sensor-based devices to prevent falls in adults and elderly in hospital environments. Additionally, this study describes aspects related with technical development of the sensors and the characteristics of their implementation identified in each of the studies.

### 2.2 Design

A systematic review and meta-analysis of the literature were performed. The data analyzed in this study were extracted from previously published studies, and therefore ethical approval was not necessary.

### 2.3 Search methods and selection criteria

This study included randomized controlled clinical trials (RCT) in which hospitalized patients were allocated to wearing sensors (portable or adhered to the bed, chair, or other elements) used by the Portuguese and English contain information related to the sensor's technical aspects. It included studies in which participants were hospitalized adults in any service of care, diagnosed with any medical or surgical condition. The intervention under study was defined as the use of movement-change sensors. during hospitalization (like from recumbent position to assuming a seated position; from seated position to standing and walking); monitored during patient hospitalization, implemented with the aim to prevent patients’ falls. Existing literature was explored on sensors used in health services to prevent falls from May 2000 to September 2019. The search was undertaken in indexed databases, such as IEEEXplore, The Cochrane Library, Scopus, Pub Med, MEDLINE, Web of Science, and ScienceDirect.

The search terms included were those related to the hospitalized population (“hospital“ *OR* “in hospital” *AND* “elderly” *OR* “adults”); the objective of using sensors (“fall prevention” *OR* “bed fall prevention”) combined with terms from the intervention (“sensors” *OR* “accelerometer” “gyroscope” *OR* “wearable sensors” *OR* “patient monitoring”) and with the type of study design (“trial” *OR* “RCT”) (Table 1). A manual search was performed, and the databases from the gray literature were reviewed.

**Table 1:** Database search: Ovid MEDLINE(R) <2000 to 2019>.

#	Terms of search	Results from
1	hospital.mp. or Hospitals/	1,207,470
2	adults.mp. or Adult/	5,419,771
3	elderly.mp. or Aged/	3,267,949
4	2 or 3	6,748,451
5	1 and 4	536,311
6	sensors.mp.	45,294
7	accelerometer.mp.	1
8	Monitoring, Ambulatory/ or gyroscope.mp.	8,922
9	wearable sensor.mp. or Wearable Electronic Devices/	4,660
10	6 or 7 or 8 or 9	55,211
11	Randomized controlled trials.mp. or Randomized Controlled Trial/	705,304
12	clinical trials.mp. or Clinical Trial/	853,142
13	Clinical Trials as Topic/ or Randomized Controlled Trials as Topic/	338,349
14	11 or 12 or 13	1,291,990
15	5 and 10	513
16	14 and 15	108
17	fall prevention.mp.	2,391
18	16 and 17	1
19	1 and 15 and 17	7
20	16 and 17	1

The search excluded studies with interventions different to the use of sensors.

### 2.4 Study outcome

Our primary outcome was the first fall of patients during their hospitalization evaluated during the course of the research. Our study did not include the rate of falls given that not all studies included data on patient-days or hospital stay.

### 2.5 Quality evaluation

Two reviewers independently used the Cochrane Collaboration tool to assess the risk of bias in each study.^[11,12]^ This tool included an evaluation of a random sequence system to assign interventions, allocation concealment compliance, blinding of participants and staff, blinding of results assessment, existence of data of incomplete results, existence of selective reports, and other sources of bias.

Besides the prior verification list, our work also implemented the Jadad scale to score the quality of the RCT (description of allocation concealment, blinding, calculation of simple size, description of withdrawals/dropouts and follow-up percentages).^[13]^ The work also scored the study by bearing in mind. the results and soundness of the recommendations stemming from this review by using the GRADE approach.^[14,15]^

### 2.6 Abstraction of information

Two reviewers selected and identified independently all the titles and abstracts of each citation of interest. Abstracts considered potentially relevant were eligible for the full text version of the study. During the second step, the reviewers used the full text of the studies to judge their eligibility for inclusion. The characteristics from each of the included studies were registered, like the country of each study, demographic data, diagnosis of patients on admission, and hospital service where the patients were cared. Information was also obtained regarding aspects such as type of intervention and duration of the intervention, description of the control intervention, and outcomes evaluated in each study. In case of missing or incomplete information, the researchers tried to contact the authors and request further information and clarification. The available information was analyzed in case said clarifications did not exist. Any disagreement in said articles was solved through consensus between the reviewers.

### 2.7 Synthesis

The nominal characteristics of the study were described by bearing in mind, whenever possible. data from the population using averages (with standard deviations), medians (interquartile ranges), counts, and percentages of continuous or categorical variables, respectively. Analysis of the results followed the intention-to-treat principle, taking all patients randomized to each arm of the study as a denominator.

Measures of the sum of the results were calculated, generating an estimate of the grouped effect and its confidence interval (CI) of 95%, using random effects models. For our result of interest, first fall during the study period (patients suffering a fall, a dichotomous variable), we calculated relative risk (RR, 95% CI). This estimation included statistics for the principal effect and to determine the degree of heterogeneity among the studies. An effect was considered statistically significant when *P* < .05. Statistical heterogeneity was calculated using the I^2^ test. I^2^ values were interpreted as “low” when they were <40%; “Moderate” between 40% and 60%; “Substantial” between > 60% and 75%; and “Considerable” if the data is between > 75% and 100%, based on the GRADE Manual.^[16]^

Furthermore, we formulated the strength of a recommendation based on the quality of the evidence according to GRADE WORKING GROUP^[14]^ (GRADEpro) program^[15]^ in high (further research is very unlikely to change our confidence in the estimate of effect); Moderate (further research is likely to have an important impact on our confidence in the estimate of effect and may change the estimate). Low (further research is very likely to have an important impact on our confidence in the estimate of effect and is likely to change the estimate) and very low (any estimate of effect is very uncertain). All the information was analyzed by using the Review Manager 5.3 software.^[12]^

## 3 Results

### 3.1 Selection and characteristics of the studies included

**Figure 1:**
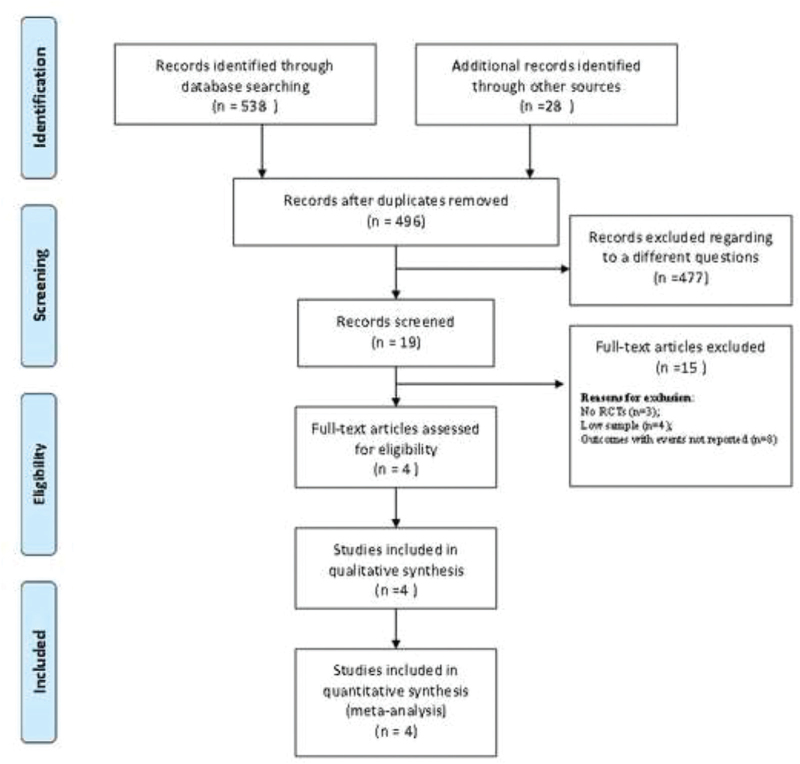
PRISMA flow chart of studies inclusion and exclusion. PRISMA = Preferred Reporting Items for Systematic Reviews and Meta-Analyses.

The initial search identified 566 records through database. After eliminating duplicates and excluding studies not related with our objective, the reviewers evaluated critically 19 potentially relevant articles. Finally, within the review, four articles were selected according to our inclusion criteria (Fig. 1 PRISMA Compliant). The studies finally included were published in English published between 2006 and 2013. The study included 29,789 patients, with total samples per study between 98 and 200 participants^[16,17]^ and between 1,839 and 27,672.^[18,19]^ The detailed description of the characteristics of the included studies can be seen in Table 2.

**Table 2:** Characteristics of studies included.

Reference, Country, Year	Gender	Mean age, years	Patients/disease	Unit of care	Outcomes	Duration	Intervention	Control
1-Wolf et al	–	–	Geriatric unitHigh risk of fall	HospitalizationSample size: N = 98	Fallers	1 year	Portable sensor located on leg. Used in two time periods: 13:00–15:00 and 20:00–06:00	Hospital standard of care for fall prevention not using sensor.
2-Sahota et al	F: 55%	84.6	Geriatric	Critical care unit, general hospitalization and geriatric unit.Sample size: N = 1,839	Fallers, and fall rate, and injuries after fall	2 years	Pressure sensors located in beds and chairs.	No use of sensors in beds and chairs
3-Shorr et al	F: 54%	59–60	Medical, neurological and surgical patients	16 Urban medical-surgical unitsSample size: N = 27,672	Fallers	1 year	Use of bed sensors and education for use and prevention measures	Conventional use of light alarm and prevention measures
4-Kwok et al	M: 54%	75–77	Stroke and dementiaHigh risk of fall patients	HospitalizationSample size: N = 180	Fallers	10 months	Sensors in beds-chairs	No use of sensors in bed or chairs

Of the four studies found, three used fixed sensors on the bed, bed-chair, or chair in the room.^[17–19]^ Only one study^[16]^ evaluated the impact of sensors (accelerometers) adhered to the patient's body (thigh with Velcro tape). The operation of the sensors adhered to the bed or bed-chair were pressure sensors that produced an alarm when the patient got up and interrupted contact with the sensor. The study by Shorr et al^[19]^ was the only one to describe the site on the bed where the sensors were placed. The intervention in the control group was standard care with a call system, using prevention measures, or no use of sensors. More details related to technical aspects and characteristics of the intervention are described in Table 3.

**Table 3:** Technical aspects.

Study, Country, Year	Sensor type and location	Communication type	Alarm type	Recorded data	Patients’ and Nurses’ perceptions	Limitations and drawbacks	Recommendations and future work
1-Wolf et al	Accelerometer.Patient's thigh.	Bluetooth	Call the nurse when detecting an attempt to get up.	Patient's movement.Hoisting detection.Messages and errors recorded.	Reliable detection and approval of its use.High acceptance by patients and nurses.Afraid by false-positive.	It was not possible to distinguish between conventional and system alarm.Evaluation of falls only when the patient was at rest.	Size reduction, waterproof, easy to disinfect and with different connection.Largest battery duration.Plans for a new clinical trial.
2-Sahota et al	Pressure sensors.Bed and chair.	Wireless	When a patient leaves the bed or chair.Provide the patient location.Pressure absence for more than 5 seconds.	A central receiver recorded all alerts in each room, which were collected by the research team.	No data	120 problems with the system.Nurses could not answer to the radiopagers rapidly.No record exists of response time or number of nurses per patient.	Nurses can answer better if devices are restrained to a minor number of patients.To prevent falls far from bed.A light alarm could be better.
3-Shorr et al	Pressure sensors.Bed, chair and toilet.Shoulders, pelvis and buttocks.Bed: 1–2 sensors.	Alarm sounds into the room, call at the nursing station.	When a patient leaves the bed, chair or toilet.Pressure absence for more than 4 seconds in bed, immediately in the chair and toilet.	Hoisting detection.Sensor contacts.	No data	Patients fell immediately alarm sounds.False alarms produce fatigue to staff.The trial was conducted only in one hospital; it might be user's contamination.	Hospitals should moderate expectations that their use will provide a simple and cost-effective solution to the problem of falls.Alarms with infrared sensors.
4. Kwok et al	Pressure sensors.Hospitalization Bed-chairs	Signal box and a wall-mount inlet point.	Pressure absence for more than 2 seconds.Intermittent light at the patient's room and a sound at the nurse station.	Loss of pressure contact.	No data	Sensors had to turn off when nurses attended patients.A large proportion of patients in EG weren’t monitored with the sensor.	The negative result of the study does not exclude the possibility that the pressure sensor can make a significant contribution to a restriction reduction program.

### 3.2 Evaluation of the quality of the studies

**Figure 2:**
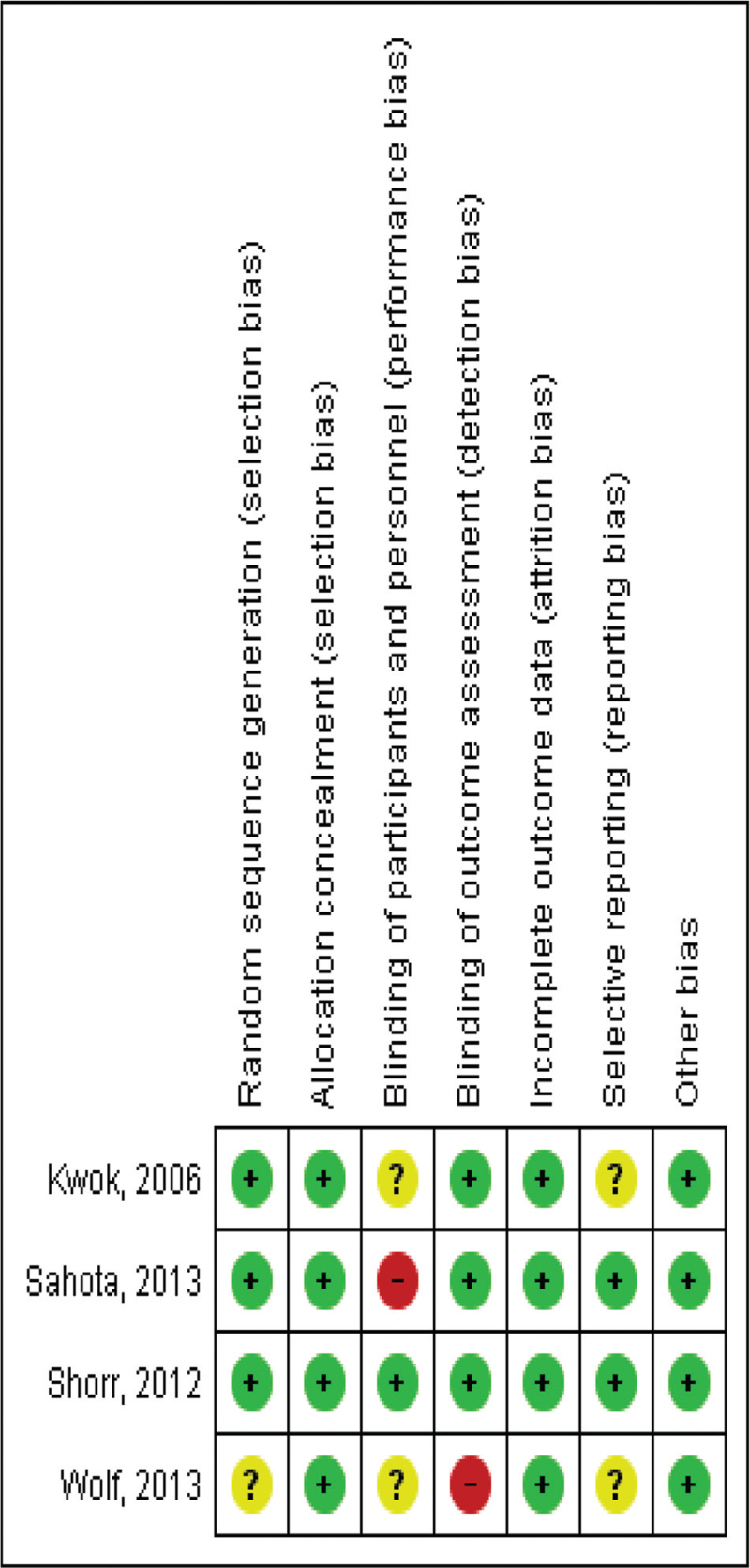
Risk of Bias evaluation (Cochrane Collaboration toll).

All the studies used a randomization system, but only three described it using random numbers.^[16–18]^ One study randomized units (clusters).^[19]^ Assignment of participants or of the units was carried out in all the studies and the manner is described (blocks, external call center, sequence of random numbers, and sealed envelopes). In general, three studies report blinding information,^[17–19]^ but the three studies reported having blinded to the researchers and the evaluators of the results. Follow-up was reported complete in all the studies and was above 90%.^[16–19]^ The study results did have a moderate estimate according to GRADE; hence, it is unlikely that the additional research changes the confidence in the estimation of the effect (See details Table 4 and Fig. 2).

**Table 4:** Quality assessment checklist (Jadad scale tool).

Author	Randomization Reported?	Random sequence generation? (selection bias)	Allocation Concealment (selection bias)	Blinding reported? Who is blinded? (performance/ detection bias)	Withdrawals/ dropouts described?	Percentage of Follow up (Attrition bias)
1-Wolf et al	Yes	Yes	By blocks	No	None reported	100
2-Sahota et al	Yes	YesBy random numbers	Yes.Central web unit at the university.	Yes,Researchers and analyst	5.6% in intervention group	93%
3-Shorr et al	Yes	Yes.By random numbers	By independent statistician	Yes	None reported	100
4-Kwok et al	Yes	YesBy random numbers	Yes.Sealed envelopes.	Yes	None reported	100

### 3.3 Primary outcomes

**Figure 3:**
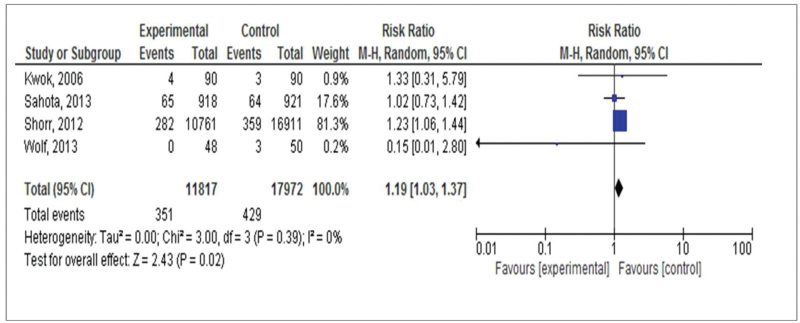
Forest plot of comparison: use of sensors in hospitalization versus conventional care intervention for fall prevention including all studies, outcome: first fall during hospitalization.

The result, first fall of hospitalized patients, was evaluated in four studies including,^[16–19]^ three evaluating the benefit of using sensors located in beds or chairs and one evaluating the benefit of portable sensors) among 29,789 patients. A total of 351 (3%) patients fell among 11,817 patients assigned to the intervention group, compared with 429 (2.4%) patients who fell among 17,972 patients assigned to the control group (RR estimation 1.19, 95% CI 1.03, 1.37, *P* = .02) (Fig. 3).

**Figure 4:**
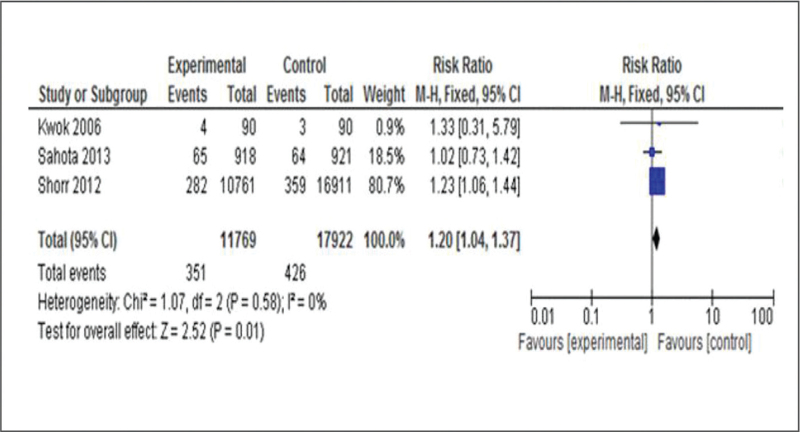
Forest plot of comparison: use of sensors in bed and chairs in hospitalization versus conventional care intervention for fall prevention excluding Wolf et al study outcome: first fall during hospitalization.

The analysis was also performed excluding the study by Wolf, which was the only one to evaluate the impact of portable sensors in fall prevention in-hospital. This result was evaluated in a total of 29,691 patients. In this case, a total of 351 (3%) patients fell among 11,769 patients assigned to the intervention group, compared with 426 (2.4%) patients fell among 17.922 patients assigned to the control group (RR estimation, 1.20; 95% IC 1.04,1.37, *P* = .02) (Fig. 4). It was identified a risk of damage in five patients per every 1000 exposed (from 1 more to 9 more).

## 4 Discussion

This systematic review and meta-analysis identified a 20% increase in the risk of falling in hospitalized elderly patients exposed to sensors installed on the bed and chair, compared with the control group. Given the size of the general estimation of the effect and the consistency of the results, we consider these results are important to call attention not only to the caregivers in hospitals caring high risk patients to fall and specialized centers in the aging, but also of hospital evaluators of these technologies and bioengineers. This evidence states a low efficacy of sensors installed in beds and chairs to prevent falls in hospitals.

Although hospital sensors are adjuvant in the medical/nursing care plans and do not replace them, its function is alert health care providers about the risk of fall according to their programmed sensitivity and specificity. The sensors identified in the studies included in this meta-analysis failed in predicting falls in real time. Because the sensors are placed on beds or chairs [are static] and are activated with the pressure of the hand exerted by the patients on the devices; these do not offer a safe and predictive warning if the patient moves away from the sensor. On the other side, once the alarm stops, it can be interpreted as if one risk patient had stopped moving, existing the chance of fall related with mobility.^[20]^ If sensors are not programmed adequately to identify changes in the movements of broad spectrum, possibly prior to risk patients getting up, they are not able to capture true and false positives, reducing in this way their capacity of prediction [positive or negative].^[8,20]^

Similar studies to ours, aimed at assessing technologies to prevent falls, as the systematic review and a meta-analysis conducted by Cameron et al^[7]^ also reported no benefit in preventing falls in patients using sensors compared with those who did not use sensors. This study included three studies (one that we excluded for low quality, and two similar to our study) and did not include independent studies evaluating portable sensors. Another systematic review carried out by Montesino et al^[8]^ performed only a description of specific factors and properties of portable sensors to enhance their predictive capacity for falls and did not evaluate the effectiveness of the sensors. The systematic review by Bet et al^[20]^ sought to describe the technical aspects related with sensitivity and specificity of sensors according to the placement site and type of sensor.

The recommendations obtained from these studies highlight the importance of portable sensors, based on their capability to identify broad changes in movement, velocity, and acceleration of the individuals who used them as well as sensitivity and specificity in detecting individuals who perform changes in movement prior to falling in real- time.

Although we identified no other clinical trials in the literature, unlike the study by Wolf et al^[16]^ comparing the effectiveness of using portable sensors during the hospitalization period, we found one study performed by Gordt ^[21]^ that explored the impact of using portable sensors among adults, but it was performed in patients living in the community. This study showed a positive effect on the re-training of the functional capacity of participants and these findings may be explored in future research evaluating efficacy of portable sensors in hospital.

These would have to overcome the limitations related to methodological aspects, such as the failures in the sensor's capacity to discriminate between true and false positives (sensitivity and specificity), and to identify the impact of the use of the sensors in individuals who may benefit most from their use and to determine the schedule for better outcomes.

Finally, future studies must reveal the complete technological details of the building of the sensors. that may be of interest to bioengineers for the improvement of sensors in hospital settings.

Regarding the quality of the methods evaluated, all studies included, in general, showed a moderate risk of bias according to GRADE. Because of the type of intervention studied (evaluation of a sensor), it was difficult to blind the patients and nursing staff. Nevertheless, most of the studies reported strategies to manage this bias by blinding the information and data for analysts and investigators. In future studies, sensors can be blinded for patients structuring placebo sensors or by developing cluster studies. Only one clinical trial evaluated the use of portable sensors, but the sample of patients was small with a weight of 1% of the general estimate,^[16]^ which is not conclusive showing no significant differences between the groups. These methodological aspects must be kept in mind during future clinical trials. Likewise, the importance of evaluating portable sensors should be proposed as adjuvant in hospital care, which must be based on individualizing care.

The use of devices of this type must be immersed within a comprehensive practice guide directed and coordinated by a multidisciplinary group, based on evidence of effectiveness to prevent falls in adult and elderly adults.

## 5 Conclusion

This study highlights the increase in falls in older adults hospitalized using movement sensors placed on the bed or chair of their rooms and explores the methodological aspects that must be overcome in the future research.

## Acknowledgments

Epidemiologist, Dr. Juan Carlos Villar C, PhD, MSc.

## Author contributions

**Conceptualization:** Olga L. Cortés, Hillary F Piñeros, Pedro Antonio Aya.

**Formal analysis:** Olga L. Cortés, Hillary F Piñeros.

**Investigation:** Olga L. Cortés, Hillary F Piñeros, Pedro Antonio Aya, Jefferson Sarmiento, Indira Arévalo.

**Methodology:** Olga L. Cortés, Hillary F Piñeros, Jefferson Sarmiento.

**Project administration:** Olga L. Cortés, Indira Arévalo.

**Resources:** Indira Arévalo.

**Software:** Hillary F Piñeros.

**Supervision:** Indira Arévalo.

**Validation:** Hillary F Piñeros.

**Visualization:** Indira Arévalo.

**Writing – original draft:** Olga L. Cortés, Hillary F Piñeros, Pedro Antonio Aya, Jefferson Sarmiento.

**Writing – review & editing:** Olga L. Cortés.
